# Quasi-Optical Four-Port Acoustic Filters Based on NEMS Coupled Beam Arrays

**DOI:** 10.3390/mi15101257

**Published:** 2024-10-14

**Authors:** Richard Syms

**Affiliations:** EEE Department, Imperial College London, Exhibition Road, London SW7 2AZ, UK; r.syms@imperial.ac.uk; Tel.: +44-207-594-6203

**Keywords:** MEMS, NEMS, directional coupler, directional coupler filter

## Abstract

Theoretical models are presented for quasi-optical four-port acoustic devices based on NEMS-coupled beam arrays. Analogies with coupled mode devices in microwaves, ultrasonics, optics, and electron wave optics are first reviewed, together with coupled beam filters. Power transfer between two mechanically coupled, electrostatically driven, coupled beam arrays is then demonstrated using a lumped element model, and the conditions for full power transfer are established. Four-port devices, including directional couplers and coupler filters with complementary transmission ports, are then demonstrated. Predictions are verified for realistic device layouts using the stiffness matrix method.

## 1. Introduction

Due to their inherently high Q-factors, devices based on mechanical resonance have long been of interest for signal processing [1,2,3,4]. Following the development of micro-electro-mechanical systems (MEMS) in the 1980s, polysilicon devices operating in the kHz frequency range were fabricated using lumped element masses and comb electrostatic drives [5,6,7,8]. Single-crystal silicon devices operating in the MHz range were subsequently developed using beams and parallel plate drives [9,10,11,12,13]. In each case, bandpass responses of different order were realized by coupling adjacent elements using mechanical springs. More recently, electrostatic springs have been used [14,15,16,17]. Extremely high performance was generally obtained, and applications as intermediate frequency filters have been proposed. However, nanofabrication has often been required to form the weak springs [18,19,20] and small electrode gaps [21,22,23] involved.

Despite investigation of alternative one-dimensional (1D) filter topologies [24,25,26,27] and 2D and 3D arrays [28,29,30], there has been a strong focus on two-port devices, ensuring that any signals that are not transmitted are reflected and limiting system architectures. In other fields of electrical engineering, four-port devices such as switches, matched filters, and channel-dropping filters are common. Examples include directional couplers formed from hollow [31,32,33,34,35] or planar [36,37,38] microwave waveguides, multistrip surface acoustic wave (SAW) couplers for signal processing formed in piezoelectric materials such as LiNbO_3_ [39,40,41,42,43], and integrated optical switches [44,45,46,47,48,49] and filters [50,51,52,53] for telecommunications formed in electro-optic materials such as Ti:LiNbO_3_. Similar devices have also been developed in fiber optic form [54,55,56,57,58], often for applications in sensing [59]. More recently, devices based on coupled guided electron waves have been investigated [60,61,62,63,64], and there is now considerable interest in applications in quantum computing [65].

In each case, the underlying principles are similar [33,39,45]. A coupling mechanism is provided to allow a slow, periodic transfer of power between two closely spaced, parallel, and initially identical waveguides. One of the guides (say, Guide 1) is then excited at its input, and the device length is adjusted for 100% transfer, so the power emerges from the output of Guide 2. A dephasing mechanism is then introduced to alter the properties of the two guides so that they are no longer identical. The power transfer mechanism is then ineffective, and the power emerges from Guide 1 instead. If the dephasing is made electrically controllable, the device can be a switch. Alternatively, if it follows from dispersion, the device is a channel-dropping filter. Refinements allow compensation for errors in the coupling length in switches [48] and reduction of sidelobes in filters [52].

Here, we show for the first time that similar devices may be realized by coupling together two electrostatically driven, coupled beam arrays commonly used for band-pass filtering. Although the devices proposed are larger than existing coupled beam filters, they are likely to be much smaller at MHz frequencies than equivalent SAW or microstrip devices whose size scales with wavelength. They are also likely to be smaller than LC filters based on surface mount device (SMD) packages and, due to the much higher Q-factors involved, have higher performance. In Section 2, the principles of coupled mode optical devices and coupled beam filters are reviewed, together with a simple lumped element model (LEM). In Section 3, power transfer between coupled beam arrays is demonstrated using the LEM, and the conditions for full power transfer are established. Four-port devices, including directional couplers and coupler filters, are then demonstrated. In Section 4, the results are verified for realistic layouts using the stiffness matrix method (SMM). Conclusions are drawn in Section 5.

## 2. Materials and Methods

In this section, we first briefly review the operation of existing four-port coupled waveguide devices, using the example of optical coupler-based devices. We then review the design and modeling of a mechanical coupled beam bandpass filter.

### 2.1. Optical Coupled Mode Devices

**Figure 1:**
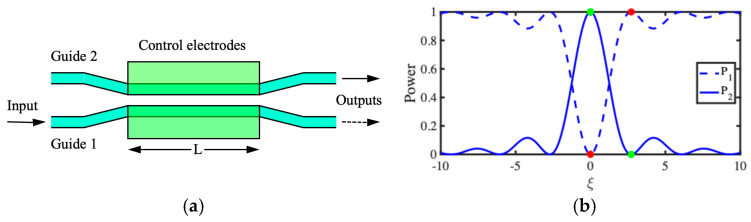
Optical directional coupler: (**a**) layout and (**b**) switch response.

Figure 1a shows an optical directional coupler, which consists of two identical dielectric waveguides of propagation constant $\beta_{0}=2\pi n_{eff}/\lambda$, where $n_{eff}$ is the effective index and λ is the wavelength. The guides are spaced apart at the input and output but run close together over a distance L at the center. Coupling is achieved by the overlap of the transverse fields in adjacent guides and can be characterized by a coupling coefficient κ.

We assume a lossless system, with unity input to Guide 1. Two different models can be used to describe the resulting behavior: a beating of the symmetric and antisymmetric modes of the composite structure [45] or a gradual transfer of power from the mode in Guide 1 to the mode in Guide 2 and vice versa [46]. In either case, the output powers $P_{1}$ and $P_{2}$ can be shown to vary with distance, as:

(1) $$P_{1}={cos}^{2}\left(\nu \right){\;P}_{2}={sin}^{2}\left(\nu \right)$$

Here, ν=κL is the coupling length. The variations are periodic, and $P_{1}+P_{2}=1$, so power is conserved. Coupling is achieved between hollow microwave guides through coupling slots [31], in SAW devices via metal strips [40], and in electron waveguides via the overlap of wavefunctions [60,61]. Despite these apparently different mechanisms, a similar periodic transfer of power is obtained in each case.

To form a switch, the coupling length is chosen so that ν=π/2 and the power emerges from Guide 2. However, the device is assumed to be formed in an electro-optic material such as LiNbO_3_ and provided with control electrodes [47]. In the arrangement shown here, application of an electric field via the electrodes will modify the propagation constants in opposite directions to $\beta_{0}\pm ∆\beta /2$, where $∆\beta =2\pi ∆n_{eff}/\lambda$ and $∆n_{eff}$ is the induced change in the effective index. The two guides are now no longer identical, and the coupling process is asynchronous. It can be shown that the output powers now vary as:

(2) $$P_{1}=1-P_{2}{\;P}_{2}={sin}^{2}\left(\sqrt{\nu^{2}+\xi^{2}}\right)/\left(1+\xi^{2}/\nu^{2}\right)$$

Here, ξ=∆βL/2 is a dephasing parameter. Figure 1b shows the variation of the two output powers with ξ. As ξ increases, $P_{2}$ decreases following a sinc-type function, falling to zero when ξ=π√3/2. At this point, all the power emerges from Guide 1. By choosing drive voltages so that outputs switch between the states indicated by colored points (known as the bar and cross states), the device can function as a switch. Any of the ports can be used as the input, and a set of switches may be combined to form an N×N switch array [49].

**Figure 2:**
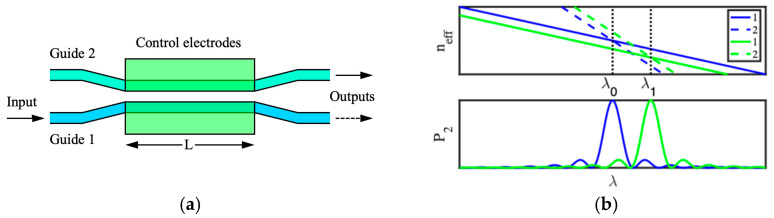
Directional coupler filter: (**a**) layout; (**b**) dispersion characteristic (upper) and spectral variation of drop-port transmission (lower) [52].

Figure 2a shows the device may be converted into a filter [52]. The layout is similar, but the guides are no longer identical and have different dispersion. The blue lines in the upper plot of Figure 2b show example variations of $n_{eff}$ with λ for the two guides, which have been engineered to intersect at a reference wavelength $\lambda_{0}$. At this wavelength, the guides are synchronous, and power transfer can again occur. If the dispersion characteristics do not intersect, the coupling may be grating-assisted [51]. Away from synchronism, power transfer is ineffective. The blue trace in the lower plot shows the spectral variation of $P_{2}$, which now approximates a sinc-function filter response. The variation of $P_{1}$ is again complementary. Application of an electric field through the electrodes can now shift the dispersion characteristics in opposite directions so that the intersection occurs at a different wavelength $\lambda_{1}$ (green lines in the upper plot of Figure 2b). The green trace in the lower plot shows the new filter response, which is correspondingly tuned.

### 2.2. Mechanical Coupled Beam Arrays

**Figure 3:**
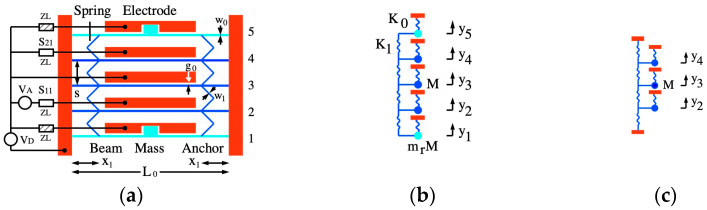
Coupled beam filter: (**a**) layout; (**b**) full and (**c**) reduced lumped element models.

We now review the operation of coupled beam arrays. We start by considering the third-order band-pass filter shown in Figure 3a, which might be fabricated by anisotropic etching and undercut of a material with a device layer thickness d on an insulating substrate. Fixed anchors are shown in red and moving parts in blue and cyan.

Operation is complicated by detuning, and a special design was introduced elsewhere to resolve the problem [66,67]. As a result, the number of beams is larger than needed and consists of 3+2=5 undercut built-in beams of length $L_{0}$ and width $w_{0}$, attached to anchors at either end. Each beam is separated from its neighbors by a distance s, and connected by a $45^{o}$ meander spring of width $w_{1}$ attached at a distance $x_{1}$ from the beam root.

Electrical connections to sources and loads are provided, as shown. Each beam has its own drive electrode, with an initial electrode gap $g_{0}$. A DC voltage $V_{D}$ is connected to all electrodes for frequency tuning. Since the electrostatic forces on the beams are equal, each will deflect without deforming the meander spring so that the electrode gap modifies everywhere to g and the resonant frequency is equally shifted. The design, therefore, ensures static synchronization. In the absence of electrostatic effects, the inner beams (blue) have identical resonant frequencies based on a combination of their own stiffness and that of the meander spring to which they are connected on either side. However, because the outer beams are only connected to this spring unilaterally, their resonances must be detuned. Although the effect is small, it may spoil a designed response involving all 5 beams. Correction is achieved by loading the outer beams with additional masses at their midpoint, recessing the electrodes to allow motion. If the masses are large enough, they can detune these beams so that they do not participate in collective oscillation near the resonance of the inner beams. The three inner beams then comprise the filter itself, and their number determines its order. Since they behave identically, these beams retain dynamic synchronization. Beam 2 is the input port and is driven by an AC source of voltage $V_{A}$ and output impedance $z_{L}$. Reflection from this port is described by the scattering parameter $S_{11}$. Beam 4 is the output and is terminated by a load, again of impedance $z_{L}$. Transmission is described by the scattering parameter $S_{21}$. Additional loads damp out-of-band motion of the outer beams; these are also taken as $z_{L}$.

### 2.3. Analytic Modeling

The device can be modeled using the LEM in Figure 3b. Here the masses M are the effective masses of the beams, and the ratio $m_{r}$ is used to describe the increased mass of the end beams. The springs $K_{0}$ and $K_{1}$ represent the effective stiffnesses of the beams and meander springs, respectively; the latter has a mass $m_{1}$, generally negligible. With electrostatic tuning, $K_{0}$ modifies to a new value ${K^{\prime }}_{0}$. The terms $y_{n}$ are the midpoint displacements of each beam. Damping is not shown but is present with a damping constant R. For in-band motion, the end masses are stationary, and the model reduces to Figure 3c.

The LEM parameters can be related to those of a real distributed structure by combining Euler beam bending theory, perturbation theory, and standard models of parallel plate actuators. For simplicity, we give only an outline here; full details may be found elsewhere (see, e.g., [9,27]). We first define the following material parameters:

- $E_{0}$ and $E_{1}$ are the Young’s modulus of the beam and spring material;
- ρ is the common material density;
- r is a distributed damping coefficient.

We then use physical dimensions to calculate the following geometric terms:

- $A_{0}=w_{0}d$ and $A_{1}=w_{1}d$ are the cross-sectional area of the beam and spring;
- $I_{0}=w_{0}^{3}d$/12 and $I_{1}=w_{1}^{3}d/12$ are the second moment of area of the beam and spring;
- $k_{1}=24E_{1}I_{1}/L_{1}^{3}$ is the stiffness of one of the meander springs.

We then use Euler beam bending theory and perturbation theory to calculate terms related to the vibration of a single unperturbed beam:

- $\omega_{1}=\beta_{1}^{4}E_{0}I_{0}/\rho A_{0}$ is the angular resonant frequency of the lowest-order mode
- $\beta_{1}$ is a constant arising from the eigenvalue equation, defined by $\beta_{1}L_{0}=\sqrt{22.37}$
- $\Upsilon_{1}\left(x\right)$ is the normalized displacement pattern of the lowest-order mode
- $\eta_{1}=0.5232$ is the ratio of the average to the maximum of ${\Upsilon_{1}}^{2}$
- $\eta_{2}=0.3695$ is the ratio of the average to the maximum of $\Upsilon_{1}$

The main LEM parameters can then be estimated as:

(3) $$M=\rho A_{0}L_{0}\eta_{2}\;K_{0}=\omega_{1}^{2}M\;K_{1}=\left(k_{1}-\omega_{1}^{2}m_{1}\right){L_{0}\Upsilon }_{1}^{2}\left(x_{1}\right)\eta_{2}\;R=rL_{0}\eta_{2}$$

We now consider actuation. The DC voltage $V_{D}$ applied to the electrodes will first deflect the beams to close the electrode gap slightly, altering the beam stiffness in the process and tuning the device. Standard theory for a parallel plate capacitor implies that:

- ${C=\varepsilon }_{0}L_{0}d/\left(g_{0}-y_{D}\right)$ is the capacitance where $y_{D}$ is a static displacement
- The normalized static displacement $y_{nD}=y_{D}/g_{0}$ is the solution to the snap-down equation $y_{nD}^{3}-2y_{nD}^{2}+y_{nD}-\gamma =0$ where $\gamma =\varepsilon_{0}L_{0}dV_{D}^{2}\eta_{1}/\left({2K}_{0}g_{0}^{3}\right)$

The solution of the snap-down equation allows C and its first and second derivatives $C^{\prime }$ and $C^{\prime \prime }$ to be found. Assuming an additional AC voltage $V_{A}$ is applied from a source with an output impedance $z_{L}$, the reduction in stiffness ΔK, effective load $Z_{L}$, and AC force $F_{A}$ are given by:

(4) $$\Delta K=\frac{1}{2}V_{D}^{2}C^{\prime \prime }\eta_{2}\;Z_{L}=({V_{D}C^{\prime }\eta_{1})}^{2}z_{L}\;F_{A}=V_{D}C^{\prime }\eta_{1}V_{A}$$

In general, the characteristic impedance $Z_{0}$ of a coupled beam array is complex, but for an infinite lossless array at resonance it has the real value:

(5) $$Z_{0R}=K_{1}/\omega_{0}$$

Here, $\omega_{0}=\surd \left(\frac{k_{0}^{\prime }+2K_{1}}{M}\right)$ is the effective angular resonant frequency, and $K_{0}^{\prime }=K_{0}-\Delta K$ is the electrostatically modified stiffness. Matching is achieved by choosing $Z_{L}=Z_{0R}$. This requires the load resistance $z_{L}$ to satisfy $K_{v}^{2}z_{L}=Z_{0R}$, where $K_{v}=V_{D}C^{\prime }\eta_{1}$. Very large values of $z_{L}$ are needed if $K_{v}$ is small, implying that electrode gaps must also be small.

### 2.4. Equations of Motion

Ignoring the shaded loads $z_{L}$, but including electrostatic tuning and damping, it is simple to show that the equations of motion for a 5-beam array with input and output ports at n=2 and n=4 subject to a harmonic drive $F_{A}=F_{0}\exp\left(j\omega t\right)$ at angular frequency ω are:

(6) $$\left\{K_{0}^{\prime }+K_{1}-m_{r}M\omega^{2}+j\omega R\right\}y_{1}-K_{1}y_{2}=0\;\left\{K_{0}^{\prime }+2K_{1}-M\omega^{2}+j\omega \left(R+Z_{L}\right)\right\}y_{2}-K_{1}(y_{1}+y_{3})=F_{0}\;\left\{K_{0}^{\prime }+2K_{1}-M\omega^{2}+j\omega R\right\}y_{3}-K_{1}\left(y_{2}+y_{4}\right)=0\;\left\{K_{0}^{\prime }+2K_{1}-M\omega^{2}+j\omega \left(R+Z_{L}\right)\right\}y_{4}-K_{1}\left(y_{3}+y_{5}\right)=0\;\left\{K_{0}^{\prime }+K_{1}-m_{r}M\omega^{2}+j\omega R\right\}y_{5}-K_{1}y_{4}=0$$

The displacements $y_{n}$ can be found by elimination, and the reflection and transmission scattering parameters $S_{11}$ and $S_{21}$ can then be extracted by standard methods.

### 2.5. Example Responses

LEM simulations were carried out for the dimensions in Table 1, which model arrays of weakly-coupled beams with resonances close to 1 MHz. The material parameters in Table 2 were chosen to model devices in (100) Si with the main and coupling beams in the <110> and <010> directions, respectively [68]. Equations (3)–(5) were used to estimate the main parameters. A mass ratio $m_{r}=1.5$ was assumed for the mass-loaded beams. The quality factor $Q=\omega_{0}M/R$ was taken as representative of a vacuum-packaged device; however, its value is unimportant, provided it is large.

**Figure 4:**
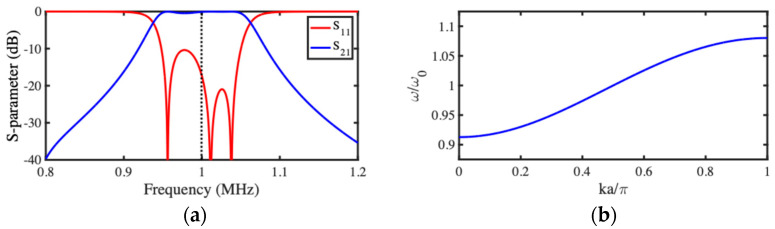
(**a**) Response of coupled beam filter; (**b**) dispersion characteristic of coupled beam array.

An AC voltage of amplitude $V_{A}=0.1\;mV$ was used for dynamic actuation. The DC voltage was first adjusted to achieve resonance at a design frequency of 1 MHz, using a value $V_{D}=2.885$ V, below the snap-down voltage of ~3.5 V, and the load impedance was then adjusted to achieve matching following Equation (5). Figure 4a shows the variation of the S- parameters with frequency. The variation of $S_{21}$ shows a passband centered at 1 MHz. $S_{11}$ is relatively high within the passband but contains a series of nulls, the central one near 1 MHz. This moderate performance is due to the approximate nature of the impedance matching relation (Equation (5)), which is strictly valid only for an infinite array at resonance. Return losses are lowered, and the central null approaches the design frequency as the bandwidth is reduced. The response of the end beams is out-of-band here but leads to a single resonance that can be damped effectively by the additional shaded loads $z_{L}$ [66].

## 3. Results

In this section, we use the LEM to show how a pair of coupled beam arrays can be combined to form four-port devices such as directional couplers and coupler filters.

### 3.1. Acoustic Wave Dispersion

We first consider how coupled beam filters can be viewed as acoustic waveguides. We note that the equation of motion for the nth element of a uniform, undamped, and undriven array such as the central mass in Figure 3b is:

(7) $$\left\{K_{0}^{\prime }+2K_{1}-M\omega^{2}\right\}y_{n}-K_{1}\left(y_{n-1}+y_{n+1}\right)=0$$

We now assume solutions in the form $y_{n}=y_{0}exp\left(-jnka\right)$, where $y_{0}$ is the amplitude, k is the propagation constant and a is the period. These solutions represent travelling acoustic waves, and substitution gives the dispersion equation:

(8) $$K_{0}^{\prime }+2K_{1}-M\omega^{2}-{2K}_{1}cos\left(ka\right)=0$$

This equation can be written in normalized form as:

(9) $$1-\frac{\omega^{2}}{{\omega_{0}}^{2}}-\kappa \;cos\left(ka\right)=0$$

Here, $\omega_{0}$ is the angular resonant frequency and κ is the coupling coefficient, given by:

(10) $$\omega_{0}=\sqrt{\frac{K_{0}^{\prime }+2K_{1}}{M}}\;\kappa =\frac{{2K}_{1}}{K_{0}^{\prime }+2K_{1}}$$

Figure 4b shows the variation in normalized frequency $\omega /\omega_{0}$ with ka over the range 0≤ka≤π, for the parameters used to calculate Figure 4a, which gives κ=0.167. The dispersion characteristic is that of a slow wave, with propagation over a finite frequency range from $\omega /\omega_{0}=\sqrt{1-\kappa }$ to $\omega /\omega_{0}=\sqrt{1+\kappa }$, corresponding approximately to the filter bandwidth in Figure 4a. The dispersion is very similar to that of magneto-inductive waves in lines of magnetically coupled L−C resonators. The effect of a finite Q-factor is to introduce loss, so the propagation constant becomes complex, and waves attenuate. The group velocity is highest, and losses are lowest at resonance, when ka=π/2.

Our assertion here is that coupled beam arrays might form the basis of acoustic coupled mode devices, provided the dispersion characteristics are suitable, the ports are terminated using matched loads, and mass-loaded end beams are provided to ensure synchronization. A simple design route is an adaptation of the device layout of Figure 3a to form broadside-coupled acoustic waveguide structures.

### 3.2. NEMS Acoustic Directional Coupler

**Figure 5:**
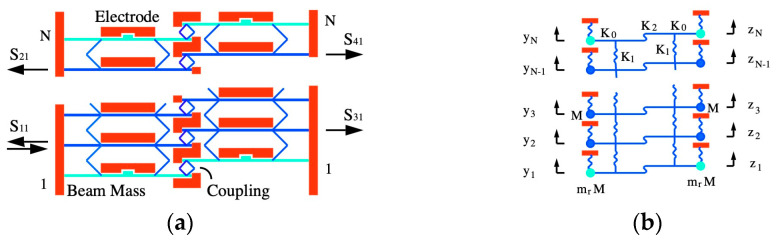
Acoustic directional coupler: (**a**) layout and (**b**) lumped element model.

We begin with an acoustic directional coupler. Figure 5a shows one possible layout, which consists of two N-element coupled beam arrays arranged side-by-side. Only a few elements are shown; the device will generally be much larger. The two arrays overlap laterally but are offset axially to allow corresponding beams in each array to be coupled together using additional diamond-shaped springs. Alternative coupling arrangements are possible, including different spring layouts and electrostatic springs. Each array is equipped with mass-loaded end beams to ensure synchronization. Electrical connections, bias voltages, and loads are analogous to those in Figure 3a but are omitted here for simplicity. The ports are numbered 1 and 2 for the left-hand (LH) array and 3 and 4 for the right-hand (RH) array. Any port may be used as an input, but here, we assume an input to Port 1 and consider the scattering parameters shown.

Figure 5b shows the corresponding LEM. Each array is modeled as shown in Figure 3b, and the terms $y_{n}$ and $z_{n}$ are the displacements of the beams in the LH and RH arrays, respectively. Again, only a few elements are shown. Corresponding elements in each array are coupled together by additional springs with an equivalent stiffness $K_{2}$, whose value may again be found by perturbation theory. Once again, insight can be obtained by considering the behavior of a large array. With no loss or driving forces, the governing equations for the nth elements can be written as:

(11) $$\left\{K_{0}^{\prime }+2K_{1}+K_{2}-M\omega^{2}\right\}y_{n}-K_{1}\left(y_{n-1}+y_{n+1}\right)-K_{2}z_{n}=0\;\left\{K_{0}^{\prime }+2K_{1}+K_{2}-M\omega^{2}\right\}z_{n}-K_{1}\left(z_{n-1}+z_{n+1}\right)-K_{2}y_{n}=0$$

Assuming travelling wave solutions $y_{n}=y_{0}exp\left(-jnka\right)$ and $z_{n}=z_{0}exp\left(-jnka\right)$ we get:

(12) $$\left\{1-\frac{\omega^{2}}{{{\omega^{\prime }}_{0}}^{2}}-\kappa_{1}\;cos\left(ka\right)\right\}y_{0}-\kappa_{2}z_{0}=0\;\left\{1-\frac{\omega^{2}}{{{\omega^{\prime }}_{0}}^{2}}-\kappa_{1}\;cos\left(ka\right)\right\}z_{0}-\kappa_{2}y_{0}=0$$

Here the resonant frequency ${\omega^{\prime }}_{0}$ and the inter- and intra-array coupling coefficients $\kappa_{1}$ and $\kappa_{2}$ are given by:

(13) $${\omega^{\prime }}_{0}=\sqrt{\frac{K_{0}^{\prime }+2K_{1}+K_{2}}{M}}{\;\kappa }_{1}=\frac{{2K}_{1}}{K_{0}^{\prime }+2K_{1}+K_{2}}{\;\kappa }_{2}=\frac{K_{2}}{K_{0}^{\prime }+2K_{1}+K_{2}}$$

Non-trivial solutions of Equation (12) (namely $y_{0},z_{0}\neq 0$) are obtained when:

(14) $${\left\{1-\frac{\omega^{2}}{{{\omega^{\prime }}_{0}}^{2}}-\kappa_{1}\;cos\left(ka\right)\right\}}^{2}-{\kappa_{2}}^{2}=0$$

Or when:

(15) $$1-\frac{\omega^{2}}{{{\omega^{\prime }}_{0}}^{2}}-\kappa_{1}\;cos\left(ka\right)\pm \kappa_{2}=0$$

**Figure 6:**
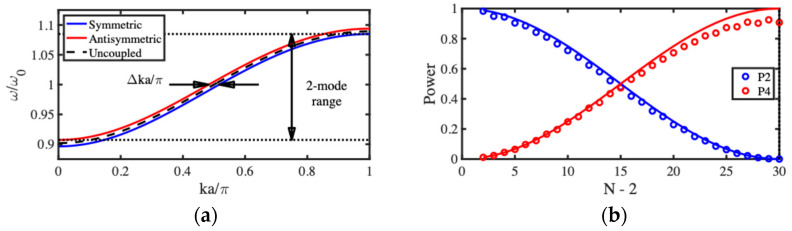
Acoustic directional coupler: (**a**) dispersion characteristics and (**b**) variation of transmitted powers with number of beams.

Equation (15) implies that there are really two dispersion equations, each corresponding to one of the two characteristic modes of the composite array. The negative sign corresponds to the symmetric mode ($z_{n}=y_{n}$) and the positive sign to the antisymmetric mode ($z_{n}={-y}_{n}$). The blue and red lines in Figure 6a shows the dispersion characteristics of the two modes for the same parameters as before, assuming in addition that $\kappa_{2}/\kappa_{1}=\pi /30$. The two curves have similar dispersion characteristics, but these are shifted in opposite directions from the uncoupled characteristic (black dashed line). As a result, each mode occupies a slightly different bandwidth, but both modes exist together over much of the frequency range. This range reduces as $\kappa_{2}/\kappa_{1}$ increases, implying that acoustic coupled mode devices are likely to be weakly coupled and consequently large.

Within the two-mode range, injection of power into one array involves excitation of both modes with equal amplitude. Because of their different dispersion characteristics, the modes will travel at different velocities, accumulating a phase difference Δka per element and beating as they go. We would then expect a periodic exchange of power between the arrays, analogous to Equation (1). When $\omega ={\omega^{\prime }}_{0}$, Equation (15) reduces to:

(16) $$cos\left(ka\right)=\pm \kappa_{2}/\kappa_{1}$$

If the RHS is small, we then obtain:

(17) $$ka\approx \pi /2\pm \kappa_{2}/\kappa_{1}$$

Ignoring the end-beams, the modes will be in antiphase with each other when:

(18) $$2\left(N-2\right)\kappa_{2}/\kappa_{1}=\pi$$

At this point, all the power injected into the LH array will have been transferred to the RH array.

The alternative coupled-mode viewpoint involves assumption of solutions $y_{n}=\eta_{n}exp(-jnka)$ and $z_{n}=\zeta_{n}\exp\left(-jnka\right)$, where $\eta_{n}$ and $\zeta_{n}$ are slowly-varying amplitudes. Substituting these solutions into Equation (11) and making use of the dispersion equation we can then obtain:

(19) $$K_{1}\left\{\left(\eta_{n+1}-\eta_{n}\right)\exp\left(-jka\right)+\left(\eta_{n-1}-\eta_{n}\right)exp(jka)\right\}+K_{2}\zeta_{n}=0\;K_{1}\left\{\left(\zeta_{n+1}-\zeta_{n}\right)\exp\left(-jka\right)+\left(\zeta_{n-1}-\zeta_{n}\right)exp(jka)\right\}+K_{2}\eta_{n}=0$$

At resonance (for example), $\exp\left(jka\right)=+j$, so the coupled-mode equations simplify to:

(20) $$K_{1}\left(\eta_{n+1}-\eta_{n-1}\right)+{jK_{2}\zeta }_{n}=0\;K_{1}\left(\zeta_{n+1}-\zeta_{n-1}\right)+jK_{2}\eta_{n}=0$$

Approximating the discrete amplitudes as continuous variations $\eta \left(z\right)$ and ζ(z), where z=na is distance, we get:

(21) $$\frac{d\eta }{dz}+j\left(\frac{\kappa_{2}}{{a\kappa }_{1}}\right)\zeta \approx 0\;\frac{d\zeta }{dz}+j\left(\frac{\kappa_{2}}{{a\kappa }_{1}}\right)\eta \approx 0$$

These equations imply a gradual transfer of power from one array to the other and are well-known to have periodic solutions [33,45]. For a pair of finite arrays with N beams, with Port 1 excited, we would expect a periodic power transfer following the relations:

(22) $$P_{2}={\left|\eta \right|}^{2}={cos}^{2}\left\{\left(N-2\right)\kappa_{2}/\kappa_{1}\right\}{\;P}_{4}={\left|\zeta \right|}^{2}={sin}^{2}\left\{\left(N-2\right)\kappa_{2}/\kappa_{1}\right\}$$

Power transfer is again maximized when Equation (18) is satisfied.

To demonstrate this behavior, the response of a finite array was found by solving the coupled equations for the LEM of Figure 5b. The points in Figure 6b show the variation with N of the powers $P_{2}$ and $P_{4}$ from Ports 2 and 4 for devices with the same parameters as before and with the inter-array coupling spring stiffness chosen so that $\kappa_{2}/\kappa_{1}=\pi /60$. The full lines show the predictions of Equation (22); there is excellent agreement with the numerical data, with the difference being accounted for by propagation loss. There is a periodic transfer of power between the two transmission ports, peaking when $N-2=\kappa_{1}/\kappa_{2}\times \pi /2=30\;$ as expected. However, an equal power split (corresponding to a 3 dB coupler) is achieved when N−2=15. For these parameters, the reflected powers $P_{1}$ and $P_{3}$ are small (<2%). These results confirm that the device can act as a synchronous acoustic coupler. Performance degrades as Q  reduces or $\kappa_{2}/\kappa_{1}$ increases. In the former case, attenuation increases, and in the latter, $P_{1}$ and $P_{3}$ both rise; however, low reflections were obtained for couplers based on arrays with as few as 12 beams.

**Figure 7:**
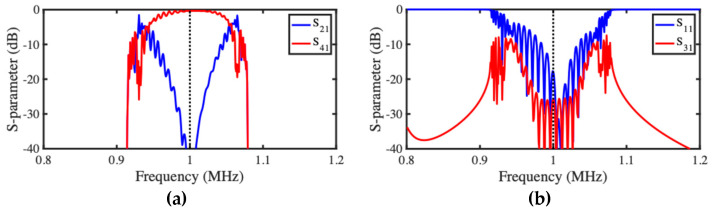
LEM model of acoustic directional coupler: frequency dependence of S-parameters for (**a**) transmission and (**b**) reflection ports.

Figure 7a,b show the frequency dependence of scattering parameters for the transmission and reflection ports, respectively, for devices based on 32-element arrays and with the same parameters as before. In this case, each array has 30 active beams, and we would expect full power transfer. This does indeed occur, and the peak value of $S_{41}$ lies at the resonant frequency (1 MHz), when $S_{21}$ is very low. Reflections are also low at resonance, and end beam motion is again out-of-band. Directional coupler operation is maintained over the two-mode range, but here $S_{41}$ varies somewhat. The explanation lies in the varying horizontal separation of the dispersion characteristics in Figure 6a, which implies that the phase shift needed between characteristic modes for 100% power transfer is only obtained at resonance. Alteration of the DC voltage applied to each array can desynchronize the coupling. However, there is little advantage in acoustic switching. Instead, we turn our attention to channel-dropping filters.

### 3.3. NEMS Acoustic Coupler Filter

**Figure 8:**
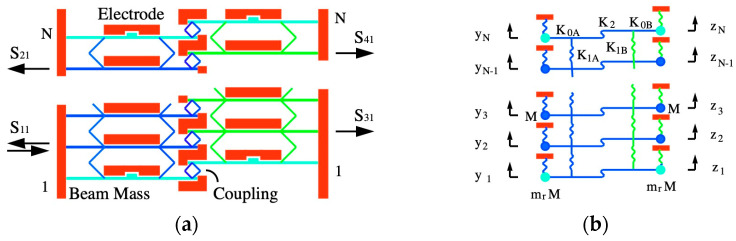
Acoustic coupler filter: (**a**) layout and (**b**) lumped element model.

To convert the mechanical directional coupler of Figure 5a into a coupler filter, all that is required is to transform the structure following the optical example in Figure 2a. Figure 8a shows the result. The beams in the LH and RH arrays now have different stiffnesses $K_{0A}$ and $K_{0B}$, while the coupling springs have different values $K_{1A}$ and $K_{1B}$.

The springs linking the two arrays have the original value $K_{2}$, and the LEM is then as shown in Figure 8b. In the absence of loss and driving forces, the governing equations for the nth elements in a large array are:

(23) $$\left\{K_{0A}^{\prime }+2K_{1A}+K_{2}-M\omega^{2}\right\}y_{n}-K_{1A}\left(y_{n-1}+y_{n+1}\right)-K_{2}z_{n}=0\;\left\{K_{0B}^{\prime }+2K_{1B}+K_{2}-M\omega^{2}\right\}z_{n}-K_{1B}\left(z_{n-1}+z_{n+1}\right)-K_{2}y_{n}=0$$

Assuming travelling wave solutions $y_{n}=y_{0}exp\left(-jnka\right)$ and $z_{n}=z_{0}exp\left(-jnka\right)$ we get:

(24) $$\left\{1-\frac{\omega^{2}}{{{\omega^{\prime }}_{0A}}^{2}}-\kappa_{1A}\;cos\left(ka\right)\right\}y_{0}-\kappa_{2A}z_{0}=0\;\left\{1-\frac{\omega^{2}}{{{\omega^{\prime }}_{0B}}^{2}}-\kappa_{1B}\;cos\left(ka\right)\right\}z_{0}-\kappa_{2B}y_{0}=0$$

Here the resonant frequencies ${\omega^{\prime }}_{0A}$ and ${\omega^{\prime }}_{0B}$ and the inter- and intra-array coupling coefficients $\kappa_{1A}$ and $\kappa_{1B}$ and $\kappa_{2A}$ and $\kappa_{2B}$ are given by:

(25) $${\omega^{\prime }}_{0A}=\sqrt{\frac{K_{0A}^{\prime }+2K_{1A}+K_{2}}{M}}\;\kappa_{1A}=\frac{{2K}_{1A}}{K_{0A}^{\prime }+2K_{1A}+K_{2}}\;\kappa_{2A}=\frac{K_{2}}{K_{0A}^{\prime }+2K_{1A}+K_{2}}\;{\omega^{\prime }}_{0B}=\sqrt{\frac{K_{0}^{\prime }+2K_{1}+K_{2}}{M}}{\;\kappa }_{1B}=\frac{{2K}_{1B}}{K_{0B}^{\prime }+2K_{1B}+K_{2}}{\;\kappa }_{2B}=\frac{K_{2}}{K_{0B}^{\prime }+2K_{1B}+K_{2}}$$

We now assume that with careful design we can arrange that $K_{0A}^{\prime }+2K_{1A}=K_{0B}^{\prime }+2K_{1B}=K_{0}^{\prime }+2K_{1}$ In this case, Equation (24) reduce to:

(26) $$\left\{1-\frac{\omega^{2}}{{{\omega^{\prime }}_{0}}^{2}}-\kappa_{1A}\;cos\left(ka\right)\right\}y_{0}-\kappa_{2}z_{0}=0\;\left\{1-\frac{\omega^{2}}{{{\omega^{\prime }}_{0}}^{2}}-\kappa_{1B}\;cos\left(ka\right)\right\}z_{0}-\kappa_{2}y_{0}=0$$

Non-trivial solutions of Equation (26) are obtained when:

(27) $$\left\{1-\frac{\omega^{2}}{{{\omega^{\prime }}_{0}}^{2}}-\kappa_{1A}\;cos\left(ka\right)\right\}\left\{1-\frac{\omega^{2}}{{{\omega^{\prime }}_{0}}^{2}}-\kappa_{1B}\;cos\left(ka\right)\right\}-{\kappa_{2}}^{2}=0$$

**Figure 9:**
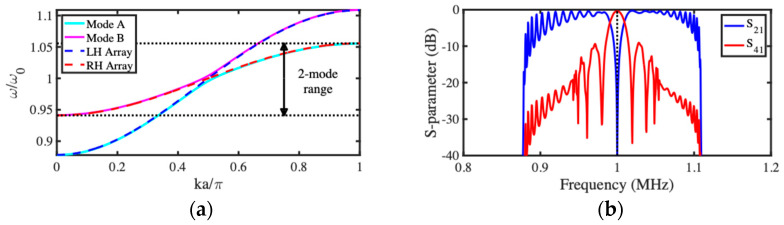
LEM model of acoustic coupler filter: (**a**) dispersion characteristics, (**b**) frequency dependence of S-parameters.

When $\kappa_{2}=0$, the equations reduce to the dispersion relations of the two arrays in isolation. The dashed lines in Figure 9a show the result obtained for the example parameters $\kappa_{1A}=0.229$, $\kappa_{1B}=0.114$, and $\kappa_{2}=0.0084$. The dispersion curves have different slopes, but cross at resonance, when synchronous power transfer must occur. The full lines show the dispersion characteristics of the composite array, which tend to the dashed-line curves away from resonance. The two-mode range is now that of the weakly-coupled RH array. The characteristic modes are now no longer fully symmetric and antisymmetric, and are therefore referred to as Mode A and Mode B. In the two-mode range, we would expect coupler filter operation. For example, if $\omega ={\omega^{\prime }}_{0}$, the two arrays are synchronous, and power transfer can occur. At this frequency, Equation (27) simplifies to:

(28) $$cos\left(ka\right)=\sqrt{\frac{{\kappa_{2}}^{2}}{\kappa_{1A}\kappa_{1B}}}$$

If the RHS is small, we then obtain:

(29) $$ka\approx \pi /2\pm \sqrt{\frac{{\kappa_{2}}^{2}}{\kappa_{1A}\kappa_{1B}}}$$

Power transfer is, therefore, now governed by the term $\sqrt{{\kappa_{2}}^{2}/\kappa_{1A}\kappa_{1B}}$, which takes the place of $\kappa_{2}/\kappa_{1}$ for couplers. Away from resonance, power transfer will be asynchronous and consequently less effective, so the device should act as a filter. A similar pair of coupled-mode equations can be derived for this case.

To demonstrate likely behavior, the response of a device based on 32-element arrays was found by solving the coupled equations for the LEM of Figure 8b. Figure 9b shows the frequency dependence of scattering parameters for the transmission ports, assuming the same parameters as Figure 9a. The overall response is band-limited by the larger passband of the LH array. Transmission from Port 4 is low outside the two-mode range but rises within it. Here, the variation in $S_{41}$ is a sinc-type function, with a narrow peak at resonance and sidelobes on either side. Within the two-mode range, the variations of $S_{21}$ and $S_{41}$ are complementary, confirming that channel-dropping has been achieved.

There is considerable freedom to achieve different filter responses. For example, the peak width (and hence the filter bandwidth) can be reduced by increasing the difference in slope of the dispersion characteristics or the number of coupled beams, in each case while maintaining the coupling length. Similarly, the filter frequency may be tuned by adjusting the dispersion characteristics so that the intersection point in Figure 9a is shifted. Depending on the coupling mechanism, these operations may be performed electrically. If very high Q-factors can be achieved, losses may be reduced sufficiently so that several filters may be arranged in series to form a filter bank.

## 4. Discussion

The results above suggest that well-known phenomena can occur in coupled sets of coupled beam arrays. However, since the predictions of an LEM may prove inaccurate, we now consider the performance achievable with realistic device layouts using the more detailed stiffness matrix method [69]. The SMM models beam networks by combining Euler beam bending theory with compatibility conditions. The aspect ratio of most MEMS suspensions validates the use of Euler theory, and transducers may easily be modeled as previously described [70]. Compared with the LEM, the main difference is the possibility of higher-order resonances. The SMM can provide results much faster than finite element methods because of the reduced number of equations that must be solved.

### 4.1. Stiffness Matrix Model

Calculations were performed using a 2D SMM solver written in Matlab^®^. The stiffness matrix K was constructed from given dimensions and material parameters, with $E_{0}$ reduced to model electrostatic detuning. Long beams were subdivided into 20 sections to ensure the accuracy of resonant frequencies. Axial, transverse, and angular displacements at each node were found for a vector of applied forces and torques (here, a point load on the actuated beam). Dynamic analysis was performed using additional mass and damping matrices. The mass matrix M was formed by combining dimensions and densities with standard relations for motions of centers of mass. The damping matrix C was modeled using Rayleigh’s method as R=aM+bK. Here, a and b are mass and spring damping coefficients, with a determined from the Q-factor and b=0. Ports were simulated by increasing the damping for these beams, using a damping coefficient determined from the load impedance $z_{L}$. Assuming harmonic forces and displacements as $\left(F,U\right)\;e^{j\omega t}$, substitution into the governing equation yields $\left(K-\omega^{2}M+j\omega R\right)U=F$. This equation was solved by inversion, and the velocity vector constructed as S=jωU. The scattering parameters $S_{11}$ and $S_{21}$ were then extracted from midpoint velocities.

### 4.2. NEMS Acoustic Directional Coupler

**Figure 10:**
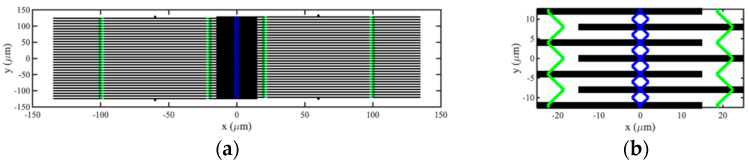
SMM model of acoustic coupler: (**a**) complete layout and (**b**) enlarged partial view.

The SMM was used to investigate the performance of devices introduced in the previous section. Figure 10a,b show the assumed layout of an acoustic directional coupler based on 32-element arrays at low and high magnifications, respectively. For simplicity, only moving parts, such as beams and coupling springs, are shown; fixed parts, such as anchors and electrodes, are omitted. Note that the aspect ratios of these figures are not 1:1. Including bond pads, the estimated device shape and size is, therefore, a die approximately 300 microns square, competitive with other technologies. The main parameters are previously given in Table 1 and Table 2. The inter-array coupling springs are of width $w_{2}$ and placed at a distance $x_{2}=0.1L_{0}$ from the beam ends, so the two arrays overlap each other by a distance $0.2L_{0}$. To tune the device, the width $w_{2}$ and DC bias voltage $V_{D}$ were iteratively adjusted to ${0.53w}_{1}$ and 2.813 V, respectively, and minor adjustments were made to the terminating impedances to maximize power transfer to the RH array at the design frequency of 1 MHz.

**Figure 11:**
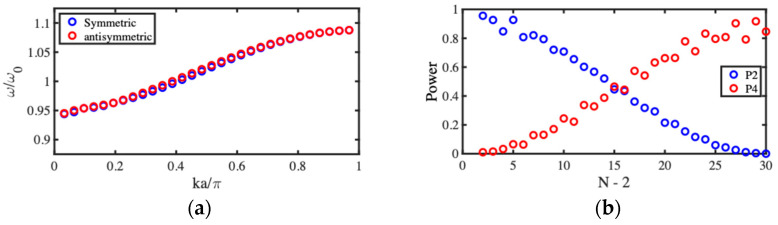
SMM model of acoustic directional coupler: (**a**) dispersion characteristics and (**b**) variation in transmitted powers with number of beams.

Dispersion characteristics were estimated by using an eigenmode solver to extract lossless resonant frequencies, retaining only those values lying in the lowest band and plotting symmetric and antisymmetric mode resonances as a function of ka/π. Figure 11a shows the result, which should be compared with the corresponding variation obtained using the LEM in Figure 6a. The SMM results are broadly similar; however, the passband is compressed at the low-frequency end. This discrepancy may be attributed to the approximations inherent in the perturbation theory used to derive the LEM.

The frequency was then fixed at resonance, losses were applied, and transmitted powers were plotted as a function of array size. Figure 11b shows the result, which should be compared with the corresponding variation obtained using the LEM in Figure 6b. The SMM results are again broadly similar; oscillatory power transfer clearly occurs, leading to full transfer at the designed value of N. Additional fluctuations may be attributed to minor impedance mismatch.

**Figure 12:**
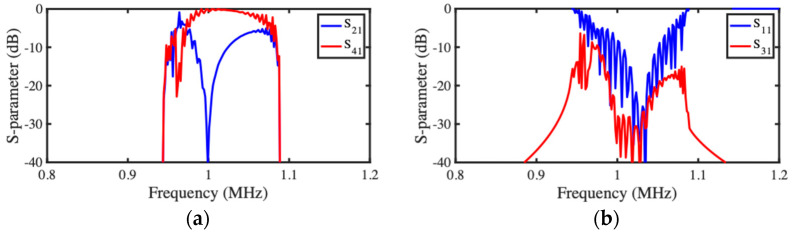
SMM model of acoustic directional coupler: frequency dependence of S-parameters for (**a**) transmission and (**b**) reflection ports.

Spectral performance was then investigated. Figure 12a,b show the frequency dependence of scattering parameters for the transmission and reflection ports, respectively. Despite some minor asymmetry caused by the passband reduction mentioned previously, qualitative agreement with the predictions of the LEM (Figure 7a,b) is again excellent, and power transfer is maximized at resonance.

### 4.3. NEMS Acoustic Coupler Filter

**Figure 13:**
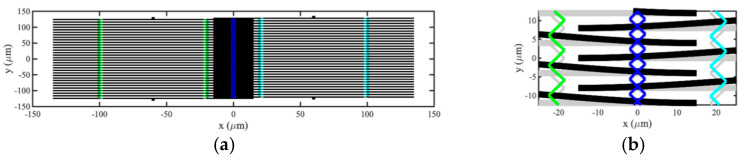
SMM model of acoustic coupler filter: (**a**) layout and (**b**) enlarged partial view after tuning.

Similarly, Figure 13a shows the layout of an acoustic coupler filter based on 32-element arrays. The main difference here is that the coupling springs in the LH and RH array now have different widths $w_{1A}=0.17\;\mu m$ and $w_{1B}=0.14\;\mu m$, indicated by different colors, while the beams have different tuning voltages $V_{DA}$ and $V_{DB}$; other parameters are as before. To tune the device, $w_{2}$, $V_{DA}$ and $V_{DB}$ and terminating impedances were adjusted to achieve maximum power transfer to the RH array at the design frequency of 1 MHz. Figure 13b shows an enlarged view after tuning, with deflections magnified.

**Figure 14:**
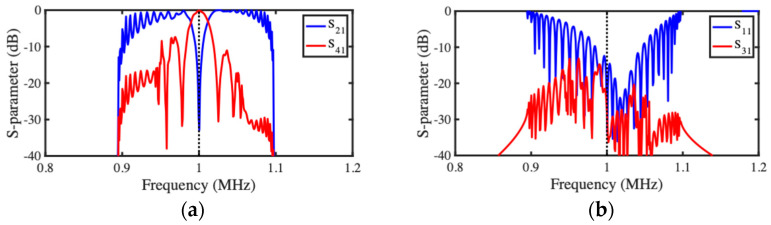
SMM model of acoustic coupler filter: frequency dependence of S-parameters for (**a**) transmission and (**b**) reflection ports.

Figure 14a,b show the resulting frequency dependence of scattering parameters for the transmission and reflection ports, respectively. Despite some asymmetry, the qualitative agreement with the corresponding LEM predictions (Figure 9b) is again excellent, implying that channel-dropping filter operation can be achieved in realistic layouts. Both models show ripples in the passband for S21 and S41. The reason is, again, imperfect matching, caused by the application of Equation (5) to this more complicated design.

Further work is required to clarify the problem and to examine whether matching can be improved by reducing inter-array coupling near the ports. Tapered coupling would have the additional benefit of sidelobe reduction. Further investigation showed that the frequency of synchronous coupling could then be varied by changing $V_{DA}$ or $V_{DB}$, allowing tunable operation.

The results above confirm that coupled-mode acoustic devices can be realized in physically achievable geometries. Similar results were obtained using alternative designs for the coupling springs, and coupling was also observed in the 3rd-order resonance band. Differences between the predictions of the idealized LEM and the more realistic SMM may be ascribed to effects not captured by the former, such as physical asymmetry and the mass of connecting springs. Layout optimization may reduce these effects, for example, by adopting a stacked rather than a broadside arrangement.

For either of the device types discussed here, the center frequency can be raised by increasing the width of the main beams or by reducing their length, and fine-tuning can then be achieved by adjusting DC bias voltages. Similarly, the filter bandwidth can be raised by increasing the width of the connecting springs or reducing their length. However, electrical adjustment of bandwidth would currently require electrostatic rather than mechanical coupling. Dimensional uniformity is clearly important. For example, it is well known from metamaterials research that individual imperfections in a lumped element waveguide will cause reflections. Multiple imperfections can then lead to cavity resonances and notches in the passband. Variations in the widths of the main beams or coupling springs must therefore be minimized, for example, to a tolerance of ±5%. The largest fabrication challenge is then presented by nanostructured features, namely the coupling springs. However, non-optical patterning processes such as sidewall transfer lithography may provide a simple method of achieving uniformity.

## 5. Conclusions

The results presented here show that quasi-optical acoustic coupled mode devices can be realized in NEMS form, merely provided two coupled-beam arrays supporting travelling waves with suitable dispersion characteristics are coupled together. However, as with other similar devices, the coupling process must be gradual to establish directionality. This investigation is preliminary and designed mainly to establish basic physical principles, but several conclusions may already be drawn.

Firstly, the layouts of acoustic directional couplers and directional coupler filters are intuitive, and simple strategies exist for voltage tuning and matching. Design can, therefore, proceed by analogy with the large repertoire of devices demonstrated and evaluated in other fields, such as ultrasonics, microwaves, guided-wave optics, and electron wave optics. Secondly, high performance requires low propagation loss and, consequently, high Q-factors, but the latter may be achieved by vacuum packaging. Thirdly, the predictions of lumped element models are verified using more detailed stiffness matrix models, so it is likely that useable performance may be realized after dimensional optimization.

Despite the above, the devices are large, and footprint reduction and performance improvement would both be required before practical use. However, if these can be achieved, applications may exist in signal separation at intermediate frequencies. To reduce overall size, the number of beams and their dimensions should be minimized. To improve performance, the bandwidth over which uniform power transfer may be achieved should also be extended by careful adjustment of coupling coefficients. Nanofabrication will likely be required to define mechanical coupling springs, and this may be a source of significant variations. However, we have already verified that electrically tuneable compensation for incorrect coupling length may be provided by methods analogous to stepped-∆β coupling [48]. Sidelobe reduction may be achieved using tapered coupling [52]. This may be more difficult to achieve since it requires a spatial variation in stiffness, but it may be possible in electrically coupled devices. Other classical device designs from microwaves or optics, such as 3 dB couplers, can be formed. As a result, coupler-based Mach–Zehnder interferometers [53] may therefore also be realizable. Finally, we note that similar phenomena are likely to occur in coupled torsional resonator arrays.

## Figures and Tables

**Table 1:** Layout parameters used in the LEM filter simulation.

$L_{0}$	$w_{0}$	d	$\alpha =x_{1}/L_{0}$	s	$w_{1}$	$g_{0}$
(μm)	(μm)	(μm)		(μm)	(μm)	(μm)
150	3	4	0.25	8	0.15	0.1

**Table 2:** Material and other parameters used in the LEM filter simulation.

ρ	$E_{0}$	$E_{1}$	$m_{r}$	Q
(kg/m^3^)	(N/m^2^)	(N/m^2^)		
2332	$169\times 10^{9}$	$130\times 10^{9}$	1.5	5000

## Data Availability

Data are contained within the article.
